# Solitary bone cyst: a comparison of treatment options with special reference to their long-term outcome

**DOI:** 10.1186/s12891-016-1012-0

**Published:** 2016-04-14

**Authors:** Frank Traub, Oliver Eberhardt, Fransico F. Fernandez, Thomas Wirth

**Affiliations:** Department of Orthopaedic Surgery, Olgahospital, Klinikum Stuttgart, Kriegsbergstraße 60, Stuttgart, 70174 Germany; Department of Orthopaedic Surgery, University Hospital Tübingen, Hoppe-Seyler-Straße 3, Tübingen, 72076 Germany

## Abstract

**Background:**

Solitary bone cysts (SBC) are benign, tumor-like lesions, which most frequently occur in the proximal metaphyseal-diaphyseal region of the humerus and femur of children and adolescents. The lack of a clear pathoetiology has impeded the development of treatment strategies. Up to date there is no consensus or official guideline for when and how treat SBC. The purpose of this study was to evaluate the effectiveness and the longterm clinical outcome of the treatment of SBC. Different techniques have been used dependant of the site of lesion, dimension, medical history and activity status.

**Methods:**

135 skeletal immature patients with a solitary bone cyst were included. A follow up of 36 months or more was available for all patients. 22 patients were treated conservatively. All the other patients had at least one surgical intervention. The following four surgical treatment modalities were used: injection of methylprednisolon acetat (steroids), intramedullary nailing (IN), IN + steroids and curettage plus bone grafting.

**Results:**

There was no significant difference between the treatment groups with respect to secondary fractures, function, pain, or complications. In the individual groups the failure rate after initial treatment was 36,6 % with steroids, 50 % with intramedullary nailing, 21,4 % with intramedullary nailing plus steroids and none in the remaining group.

**Conclusion:**

Steroid injection remains a reliable method for treating solitary bone cysts owing to its low invasiveness. To prevent fractures and allow a full weight bearing, internal fixation in combination with methylprednisolon acetat injections seems to be the most favorable in weight bearing bones.

## Background

Solitary bone cysts (SBC) also known as unicameral bone cysts (UBC) are benign, fluid-filled, single chambered tumor-like lesions. They most commonly occur in the proximal metaphyseal-diaphyseal region of the humerus and femur of children and adolescents. The absolute incidence is unknown, it is estimated that approximately 3 % of all bone tumors are SBC [1]. Since SBC show specific radiographic features on plain x-ray further imaging studies are usually not required, especially in long bones. The classic appearance is a centrally located, radiolucent, well-marginated, slightly expansile lesion of the proximal metaphysis. The growth plate is normally not affected by these expansive cysts. The cortex of the bone have a tendency to get thin and weak. This thinned cortex is an area of structural weakness and is prone for a pathological fracture. The ‘fallen leave’ sign is indicative for SBC [2]. The cyst is often asymptomatic, but pain in the affected region may be present. Often the first and only symptom is a pathological fracture following a trivial trauma [2, 3], but sometimes the initial diagnosis is made after a radiological investigation performed for other reasons.

Since the first documentation of SBC by the German pathologist Rudolph Virchow in 1876 [4], the cause remains unspecified. Therefore the evolution of a conclusive treatment targeting the source is still missing. Numerous hypotheses have been proposed to explain the pathogenesis of SBC, e.g. bone resorptive properties of the cyst lining, vascular obstruction, increase in intracavitary osseous pressure,inflammation or traumatic causes [5–10].

There is a wide selection of options to handle SBCs, including observation, decompression of the cyst (using cannulated screws or nails) instillation of bone marrow, steroids or demineralized bone matrix, mechanical disruption of the cyst (usually by curettage), structural support (e.g. flexible intramedullary nails or bone grafting) or combined approaches that address the lesion on multiple levels.

The objective of therapy is to prevent a pathologic fracture or re-fracture, promote cyst healing, and to avoid cyst recurrence. The purpose of this study was to assess the healing rates of SBC after different therapy modalities as well as the long term clinical outcome after treatment.

## Methods

The patient archive at Olgahospital Stuttgart was searched to identify all skeletal immature patients treated for SBC between 2000 and 2010. We found 164 patients (112 boys and 52 girls) who were diagnosed and treated with a SBC. The inclusion criterions for this study were: a solitary bone cyst (diagnosed by histological or radiological features) and follow up for at least 3 years or Neer classification I and skeletal maturity [2]. 29 patients were excluded, because of insufficient long-term clinical information (follow-up <3 years) or lost to follow-up. 22 patients had been managed conservatively (e.g. splint, cast), because the probability of healing was considered very high (*n* = 18), or the consent for an operation was denied. All the other patients (*n* = 113) had at least one kind of surgical intervention. The patient demographics are shown in Table 1.

**Table 1:** Patient demographics and Site of the lesion

Mean age in years	9,6 (4,2 – 16,4)	
Male/female	87/48	
Active cyst	74	55 %
Fracture before initial treatment	93	69 %
Humerus	59	44 %
Femur	49	36 %
Tibia	7	5,2 %
Fibula	5	3,7 %
Radius	2	1,5 %
Calcanues	8	5,9 %
Os pubis	3	2,2 %
Os ilium	2	1,5 %

### Surgery

All procedures were performed under general anaesthesia. Surgical procedures were the following:

- aspiration and injection with methylprednisolone (group I),
- elastic stable intramedullary nailing (group II),
- a combination of the above (group III),
- curettage followed by bone grafting with an autograft or allograft (group IV).

### Steroid injection

We used the technique of Scaglietti [9]. in a modified manner. A 11-gauge aspiration needle was inserted percutaneously to aspirate the contents of the cyst. 80 to 160 mg of methylprednisolone-acetat was inserted into the cyst. The dose of steroid ranged depending on cyst size. The patient was discharged on the same day, after the injection,. This procedure was performed three times with an interval of four to six weeks between every injection. Pathological fractures were allowed to heal for six weeks before the treatment with steroids was started. All patients treated with steroids as a primary intervention received exactly 3 injections.

### Intramedullary nailing

For intramedullary nailing we followed the classical surgical technique, as reported by Ligier et al. [11] using a retrograde nailing approach. In case of a pathological fracture, first the closed reduction of the fracture was performed. Under fluoroscopic guidance the elastic intramedullary nailing (in an ascending manner) was completed. The diameter of the nails (2.0 to 3.0 mm) was selected on the basis of the anterior-posterior radiograph and intraoperative findings according to the principles described earlier [11]. Same setup was used in the case of prophylactic intramedullary nailing.

### Curettage

Utilizing a standard approach to the different locations of the cyst, the cortex above the lesion was opened by four to six drill holes thru the cortex and using a chisel to connect the holes and remove the bony window. After the fluid was removed, the membrane of the cyst was rigorous curetted. To monitor the procedure the fluoroscope was used to assure that the curettage reached the outside of the SBC.

### Bone graft

For bone grafting either autologous bone from the iliac crest or allogenic bone (Phoenix®, TBF, France) was used and the complete cyst was packed.

### Postoperative treatment and radiological control

Postoperative treatment was aligned to the specific findings of the lesion. The therapy varied upon the site and location, size and volume, presence of fracture and displacement etc. Thus treatment (not only postoperative) ranged from simple dressings and splints to cast therapy and activity and weight bearing restrictions.

Radiographs were taken six and 12 weeks after surgery and from then on every six months. Controlled by improvement or recurrence the radiological monitoring was individually modified. All radiographs taken during the course of treatment were analyzed and the cyst volume was calculated (length by width by depth in cubic centimeters based on two orthogonal plain radiographs). SBCs are classified into active or latent according to Jaffe and Lichtenstein [7]. When the distance to the physis was less than 10 mm the cyst was classified as an active cyst. All cyst showing more tan 10 mm distance from the physis were classified as latent.

Measuring the volume of the cyst is an attempt to quantify the SBC, but normally the volume is not matched to the size of the bone. One option is to match the volume to the diameter of the (normal part of the) shaft as it was proposed by Kaelin and MacEwen as “cyst index” [12].

The Neer/Cole rating system [2, 13] was used to monitor treatment response. The radiographic results were defined as I (healed) when complete obliteration of the cyst was shown. Grade II (residual defects) was defined new bone filled the lesion, but some opaque areas were still present. Grade III (cyst visible) was defined when multilocular and lucent residue areas were visible. Grade IV (cyst clearly visible) was assigned when the cyst was unchanged after the treatment. Grades I and II were considered as good results: cyst healed; grades III and IV were considered poor results: cyst not healed or pending pathological fracture.

The primary study outcome was the cyst-healing and the secondary outcome included the function and activity, pain, subsequent fractures, and complications. Pain that persisted at least 1 year after surgery was scored as a binary variable (present or absent). Physical activity was scored on a five-level response scale (1 not restricted in daily activity/playing – 5 substantial limited in daily activity/playing).

Do determine factors influencing the healing rate, the following parameters were included into the analysis:

Sex, Age, Site, Cyst volume, Cyst status, Cyst index and Fracture before initial treatment.

### Statistical analysis

Analysis was conducted using SPSS software (version 15, SPSS Inc., Chicago, IL, USA). For the analysis of dichotomous variables between two groups, a chi-square cross tab test was performed. Values of *p* < 0.05 were considered significant. Recurrence rate was determined using the Kaplan-Meier method with treatments compared by the log rank test.

## Results

In 83 of 135 patients the diagnosis of SBC was confirmed by the pathologist. Since no material for histological examination was obtained in the 52 remaining patients the diagnosis based on the typical radiographic morphology. At presentation, 93 patients (68.9 %) had a pre-existing pathological fracture. In our patients pathological fractures significantly involved the humerus more often than the femur (*p* = 0.039). There was an equal distribution of fractures in both sex. 39 patients (28.9 %) complained of pain at the site of the lesion and in 3 patients the SBC was recognized accidentally, because an x-ray was done for other reasons.

The median follow-up time period for all patients was 57.2 (36 – 92) month. The cysts were mainly located in long bones (*n* = 122), most commonly in the humerus (*n* =59) and the femur (*n* = 49). The other locations in long bones were the tibia (*n* = 7), fibula (*n* = 5) and radius (*n* = 2). Nearly 90 % (109 of 122) of the lesions were located in the proximal part of the bone. Eight cysts were located in the calcaneus, three in the os pubis and two in the ilium.

The cyst volume found to be related to healing rate. If the cyst volume was > 84.3 cm^3^ the healing rate for the SBC was significantly reduced in comparison of all the groups (*p* < 0.03). Considered the individual group, there was no significant difference regarding the cysts size and the healing rate allocable. Size and pathological fracture was only dependent in long bones. From a size of 96.4 cm^3^, the probability of a pathological fracture was significantly increased (*p* < 0047). The volume tends to be bigger in the upper extremity compared to the lower extremity, but did not reached significance in our patients.

The cyst index at diagnosis showed a significant relation to the healing rate. The cyst index was similar in all different groups but showed differences among the locations (upper extremity average 5.6 (SD 1.8) and lower extremity average 4.8 (SD 1.3)). When the cyst index was correlated with the healing rate the reference values suggested by Kaelin [12] were used. In the upper and lower extremity a cyst index < 3.5 showed a significant lower failure rate than a cyst index > 5 - independent from the treatment groups.

Of the 135 patients, 74 had an active bone cyst at an age of 8.3 years and the other 61 had latent bone cysts at an age of 11.8 years. Patients with active cysts were significantly younger (*p* = < 0.01). In active cysts of long bones we recognized a higher failure rate. This was not significant between the different groups, nor the location in the upper or lower extremity. A list of a parameters included into the analysis is shown in Table 2.

**Table 2:** Location of the SBC in the different groups. The number in brackets shows failure of first line treatment

Clinical factors	No.	No. of healing (%)	*p*-value
Sex			
Male	87	63 (72.4 %)	n.s.
Female	48	35 (72.9 %)	
Age			
< 10 years	92	70 (76.1 %)	n.s.
> 10 years	43	28 (65.1 %)	
Site			n.s.
Humerus	59	42 (71.2 %)	
Femur	49	34 (69.4 %)	
other long bones	14	10 (71.4 %)	
others	13	12 (92.3 %)	
Volume			
< 84,3 cm^3^	50	42 (84 %)	**0.0279**
> 84,3 cm^3^	85	56 (65.9 %)	
Cyst status			
Active cyst	74	44 (59.5)	**0.0214**
Latent cyst	61	54 (88.2 %)	
Cyst index (in long bones)			
< 3.5	35	30 (85.7 %)	**0.0119**
> 5	43	25 (58.1 %)	
Fracture			
Yes	93	72 (77.4 %)	n.s.
No	42	26 (61.9 %)	

The data in boldface represents the Chi-square test and Fisher’s exact test

**Fig. 1:**
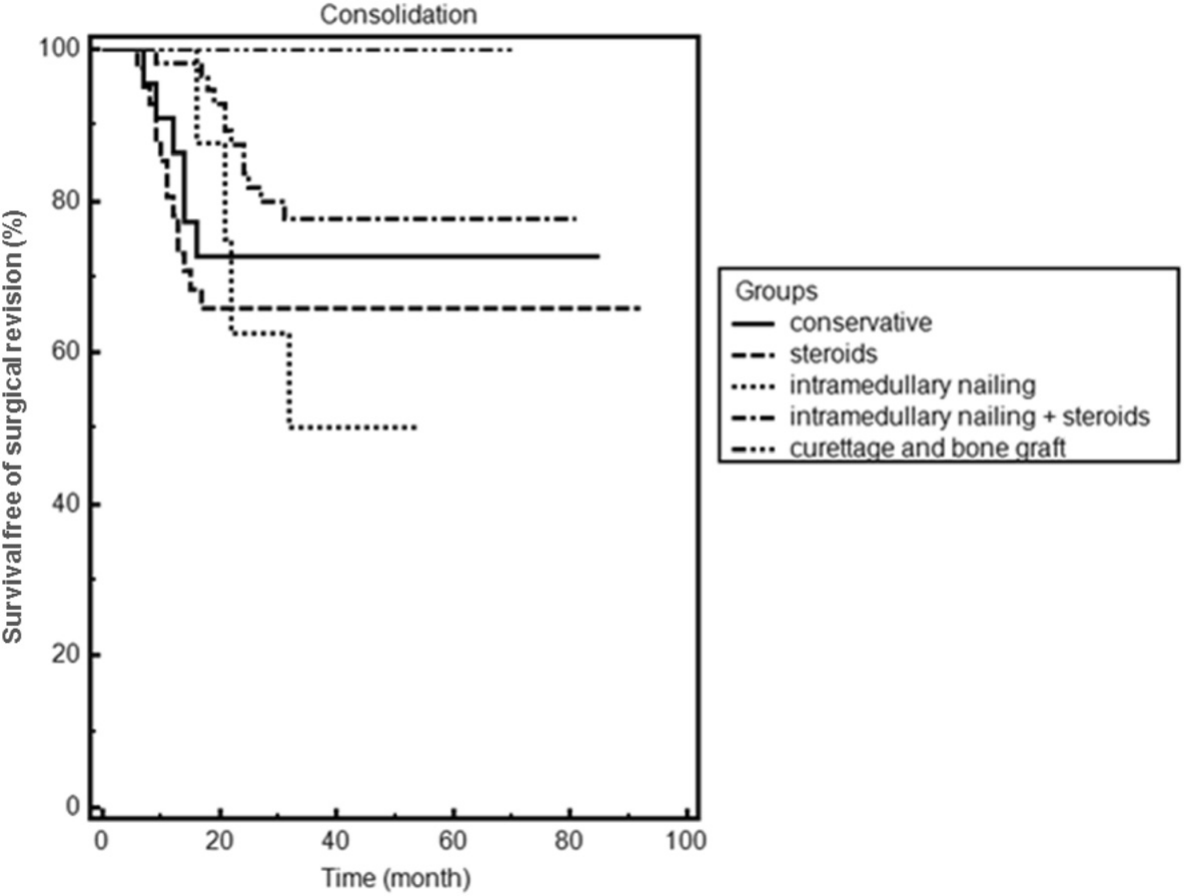
Kaplan-Meier analysis of the different treatment groups after initial treatment

The failure rate in the conservative group was 27.3 % (6 of 22). Overall recurrence rate after primary surgical therapy was 26.1 % (31/119) for all surgical procedures - recurrence being defined as requiring further surgical treatment (re-fracture, grade III/IV, limited function or ongoing pain). 19 patients from the conservative group (*n* = 5) and group I (*n* = 14) experienced a fracture after initial treatment. There were no significant associations between fractures and cyst activity, cyst area, loculation, location in the upper or lower extremity, or location within the bone. In the individual groups the failure rate after initial treatment was 36.6 % (15 of 41) with steroids, 50 % (4 of 8) with intramedullary nailing, 21.4 % (12 of 56) in the intramedullary nailing plus steroids and none in the curettage and bone graft group (see Table 3). The Kaplan-Meier analysis shows that further surgical treatment was required between 10–20 months after initial treatment (Fig. 1). Steroids and conservative treatment were associated with earlier failures than the other therapies. The failure rate was significantly higher in patients with an active bone cyst, independent from treatment and location (*p* < 0.03).

**Table 3:** The influence of different clinical factors on the healing rates

Location	Conservative	Steroids	IN	IN + steroids	Curettage + bone
	(*n* = 22)	(*n* = 41)	(*n* = 8)	(*n* = 54)	Graft (*n* = 8)
Humerus	8 (4)	20 (8)	3 (1)	27 (4)	1
Femur	3 (1)	13 (3)	5 (3)	26 (8)	2
Tibia		Tibia		1	2
Fibula	3 (1)	2 (1)			
Radius	1	1			
Calcaneus	6	1			1
Os pubis	1	1 (1)			1
Os ilium		1			1

After the surgery occasional pain, numbness, or tingling was reported on the first visits (up to 6 month after last treatment), there was not significantly different among all the groups. Regarding the function there was also no difference between the treatment groups. Function was persistently very good in all the patients (not restricted in daily activity). We did not see a cyst recurrence after the cyst was completely healed (Neer Grade I). At the time the patient reached skeletal maturity the majority of the SBC were healed completely, only 22 % of the SBC were classified Grade II. Not one of the patients reached skeletal maturity with a cyst graded III or IV.

The first treatment choice was not successful in 37 patients, and all patients underwent an invasive intervention. Three out of the 37 patients needed a third operation. Five patients received a second course of steroid injection (3× injections). One out of these five needed a third intervention. In nine patients the IN had to be change, because the nails did not bridge the cyst. These cysts healed without any further treatment. 17 patients were retreated with intramedullary nailing plus steroids, in two patients the cyst failed to heal after this retreatment. In six patients a curettage and bone grafting was performed as second line treatment, all of them healed completely. In all patient (*n* = 3) where the second line treatment failed, a curettage with bone graft was performed.

Seven patients developed complications after invasive treatment; one patient was treated for a donor-site infection after autologous bone grafting. Three patients had a growth disturbance with a mild deformity of the shaft, and two patients showed a limb shortening with epiphyseal arrest. In one patient a femoral head necrosis was observed - in this boy the cyst did not respond to the steroid injection and no response was noted after intramedullary nailing, thus a curettage and bone grafting was performed. This is the only patient were the function was not restored completely 2.5 years after the last treatment. He still has some limitation walking long distances and running or jumping.

## Discussion

Whenever a child with a SBC is presented to a orthopedic surgeon the decision whether to observe or treat has to be made. There is no consensus or official guideline for when to treat a SBCs. If the decision is made to treat the child with a SBC the demanding questions is, how to treat. Currently, there is also no covenant on a reliable exemplary therapy. Up to date there is an only one multi-center randomized trial published, comparing intralesional steroid injection and bone marrow injection [13]. The indications for an invasive treatment should consider prevention of pathologic fracture, reduction and stabilization of fractures, prevention of prolonged activity restriction and the consolidation of the cyst. In the literature some reliable methods to calculate the fracture risk are reported [14, 15]. Thus our aim was to share our therapy experience and help to identify reliable treatment for SBC.

Like the most retrospective studies this study has certain limitations, including inherent selection bias. Indications and treatment selection were based on clinical criteria and physician discretion rather than objective criteria. However, we believe the high number of patients analyzed compensate for any such inconsistencies. Nonetheless, our retrospective study of 135 patients with a SBC having an average follow-up of ~5 years is one of the largest in the literature. In our patients we had significantly more humerus than femur fractures. This finding is in concordance with previous studies [5, 16]. It is assumed that the lack of weight load in the humerus enable the cyst to grow unrecognized until the structural characteristics of the bone are limited and subsequent a pathological fracture occurs. Since the long bone of the lower extremity withstand a considerable weight load pain as a first symptome is recognized.long before a mechanical threat happens. The cyst index, which was increased in the humerus compared to the femur support this hypothesis too.

After a course of three consecutive intralesional injections of methylprednisolon acetat the failure rate was 36,6 % in our patients. In comparable trials the rate is between 32 and 24 % [3, 17, 18]. In a previous study from our institution including 71 patients with SBC the failure rate was similar ~30 % [16]. Although Scaglietti et al. [9] have reported doses up to 240 mg, we are not aware of any dose–response curve that suggests higher doses are more effective. The study by Wright et al. [13] revealed that steroids are the only evidence based therapy.

To achieve higher rates of success with one single intervention, some authors advocated injection of bone marrow, together with multiple perforations of the cyst wall to re-establish the continuity between the cavity of the cyst and the adjacent normal marrow [8, 13]. This strategy makes it difficult to separate the effects of mechanical disruption from those produced by the osteogenic capacity of bone marrow injected into the cyst. In another report of 79 patients where steroid injections were compared with injection of bone marrow, no advantage of one or the other method could be shown [19]. Referring to the only randomized prospective clinical trial steroid injection still showed a significantly better outcome than bone marrow [13].

The intramedullary nailing of long bones is not a new concept [20]. Some studies reported and success rates between 75 and 100 % [21, 22]. Using this technique has the advantage of a minimal invasive procedure, immediately stability and continuous decompression. Since there is no further cortical impairment (as in e.g. curettage), hospital stay and return to normal activity are positively affected. Moreover, this technique can easily be combined with other procedures during one intervention, such as injection of steroids. The success rates in the presented study of 50 %, resp. 79.6 % are consistent with those reported in the literature. The relatively high failure rate of 50 % can be explained with the growth of the children. The nails did not bridge the cyst in 3 of 4 cases during the healing course, and an exchange of the intramedullary nails was necessary. This hypothesis is supported by the proportional late consequential operations. We did not find significant differences of the treatment according to the location (upper vs. lower extremity). In our experience the combination of intramedullary nailing and a course of steroid injection showed the best clinical result and is our preferred treatment option in the case of non obliteration of the cyst or in weight bearing long-bones.

Curettage and disruption of the cyst lining combined with bone grafting has been considered as the gold standard for many years [2]. This procedure provides enough osteogenic stimulus to enhance healing [16, 23]. Nevertheless, there is a need for less invasive strategy, considering the benign and self-limited nature of the lesion [24]. In a small study percutaneous curettage alone showed to be superior to intralesional injections and thus this approach was favored by the authors [25]. Studies reporting the efficacy of bone substitutes are limited [17]. Only few clinical studies are found in the literature. The recovery rates vary between 66 % up to 100 %. But most of these studies have a lack of follow-up >12 month after diagnosis [6]. In our study, patients initially treated by curettage with a combined bone grafting (group IV) no recurrence was observed. We assume that these good results can be explained on the one hand by the location of the SBC and the mechanical stress and on the other hand to the age of the patient (>11 years).

In this study we were able to offer long-term clinical outcome for conservative and surgical treatment of SBC. Our results confirm the satisfactory overall long-term outcome. Focusing on short-and mid-term surveillance a substantial rate of recurrence for all different treatment strategies could be detected. Long-term follow up of more than 24 months is rarely conveyed in many other studies. We not only focused on the radiographic appearance, but also included, pain, function and re-fracture rates. Considering the assumed long-term success of SBC therapy, invasive strategies have to be carried out as cautious as possible. In one study with a 7 year follow-up the residual cyst persist into early adulthood, but no further fractures were detected even when the healing was graded III or IV [26].

## Conclusion

We believe that the invasiveness of the procedure must be contrasted by the need for repeated treatment (e.g. steroids). These factors must be balanced against risk of fracture and probability of success rate. Small, non symptomatic and latent SBC (particular in the calcaneus or the pelvic bones) can be observed and if necessary, therapy with injection of steroids can be started. Since we did not have any fractures in our group III we recommend internal fixation and the injection of steroids in long bones with a mechanical load to reduce fracture rate and to expedite the recovery of the child. In the upper extremity the cyst volume, the cyst index and the activity level should be considered when making a recommendation. The treatment options of conservative versus steroids injection versus fracture fixation and steroids should be discussed with the patient and the parents.

### Ethics and consent to participate

An institution approval for this investigation was granted and all investigations were conducted in conformity with ethical principles of research. Informed consent for evaluation and anonymized assessment was obtained from all patients before treatment was started. Since all data could be obtained without further treatment or monitoring procedures the ethical committee (University of Tuebingen, Germany) waived the approval, this procedure is in accordance with the European regulations and the requirements for observational studies in Germany.

### Consent to publish

Not applicable.

### Availability of data and materials

All the data supporting your findings is contained within the manuscript.

## Abbreviations

IN: intramedullary nailing
SBC: solitary bone cysts
Steroids: methylprednisolon acetat
